# The Role of Mast Cells in Tuberculosis: Orchestrating Innate Immune Crosstalk?

**DOI:** 10.3389/fimmu.2017.01290

**Published:** 2017-10-17

**Authors:** Karen M. Garcia-Rodriguez, Anu Goenka, Maria T. Alonso-Rasgado, Rogelio Hernández-Pando, Silvia Bulfone-Paus

**Affiliations:** ^1^Manchester Collaborative Centre for Inflammation Research, Faculty of Biology, Medicine and Health, School of Biological Sciences, Manchester, United Kingdom; ^2^Faculty of Science and Engineering, School of Materials, University of Manchester, Manchester, United Kingdom; ^3^Departamento de Patología Experimental, Instituto Nacional de Ciencias Médicas y Nutrición “Salvador Zubiran”, Mexico City, Mexico; ^4^Division of Musculoskeletal and Dermatological Sciences, Faculty of Biology, Medicine and Health, University of Manchester, Manchester, United Kingdom

**Keywords:** *Mycobacterium tuberculosis*, mast cells, degranulation, extracellular traps, granuloma, infection

## Abstract

Tuberculosis causes more annual deaths globally than any other infectious disease. However, progress in developing novel vaccines, diagnostics, and therapies has been hampered by an incomplete understanding of the immune response to *Mycobacterium tuberculosis* (*Mtb*). While the role of many immune cells has been extensively explored, mast cells (MCs) have been relatively ignored. MCs are tissue resident cells involved in defense against bacterial infections playing an important role mediating immune cell crosstalk. This review discusses specific interactions between MCs and *Mtb*, their contribution to both immunity and disease pathogenesis, and explores their role in orchestrating other immune cells against infections.

## Introduction

Tuberculosis (TB) is the world’s major infectious disease killer, accounting for 1.4 million deaths in 2015 (1). Progress in developing vaccines, diagnostics, and therapies has been hampered by an incomplete understanding of the immune response to the causative pathogen, *Mycobacterium tuberculosis* (*Mtb*).

Following entry of *Mtb*-containing droplets into the airways, bacilli are initially phagocytosed by alveolar macrophages (AMφ), providing a comfortable niche in which *Mtb* can reside, replicate, and evade immune cell detection (2). Mycobacterial pathogen-associated molecular patterns engage pattern recognition receptors (PRRs) to trigger signaling pathways, resulting in the release of various chemokines and cytokines and the recruitment and activation of immune cells (3). This process results in the internalization of mycobacteria by dendritic cells (DCs), which migrate to lymph nodes where they polarize naïve T cells to antigen-specific Th1 effector cells in an IL-12-dependent manner (4, 5). IFN-γ produced by Th1-polarized T cells activates mycobactericidal mechanisms in AMφ (6). Various immune cells are sequentially recruited to the sites of infection; neutrophils in the earliest stages as well as T cells, NK cells, and fibroblasts. These surround the infected AMφ to form a mycobacterial granuloma (2, 7), which acts as a “physical barrier” limiting bacillary dissemination. However, chronic granulomas also promote *Mtb*’s intracellular survival and impair elimination, resulting clinically, in latent TB disease (2). One-third of the world’s population is latently infected with *Mtb* and between 1 and 10% will develop progressive TB disease following “reactivation” of infection in later life (8). The mechanism of TB reactivation is unclear as yet, however, it is suggested that a failure of granuloma maintenance may be the cause (9). Thus, the granuloma is the result of a non-efficient immune control that will eventually progress to a chronic infection, rather than mycobacterial clearance.

Mast cells (MCs) are tissue resident cells strategically located in mucosal tissues (10) and are among the first cells to come in contact with pathogens (11). MCs contribute to bacterial immunity through multiple mechanisms such as bacterial recognition, activation, recruitment of immune cells to the site of infection, release of inflammatory mediators, and direct bacterial killing by extracellular traps (ETs). However, their main role may be orchestrating other immune cells against infections (11–14).

**Figure 1:**
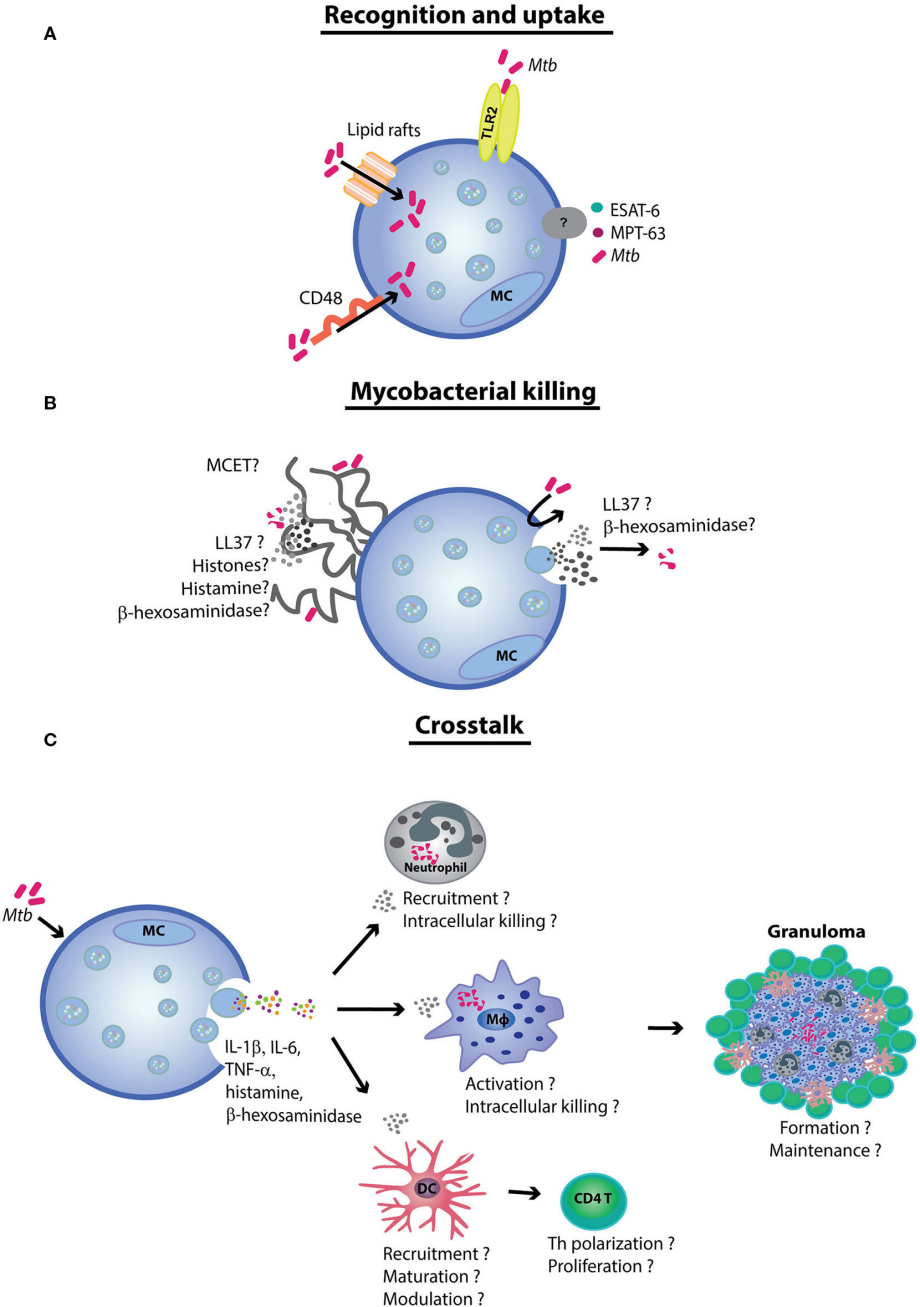
The role of mast cells (MCs) in tuberculosis. **(A)** MCs recognize *Mycobacterium tuberculosis* (*Mtb*) *via* the TLR2 and CD48 receptors. The latter also contributes to *Mtb* uptake. Although the uptake process remains yet unclear, mycobacteria have been demonstrated to be internalized by lipid rafts. **(B)** *Mtb* and the mycobacterial antigens early secretory antigenic target 6 (ESAT-6) and MPT-63 induce MC degranulation and cytokine release. It is likely that *Mtb* exposure induces antimicrobial peptide secretion and mast cell extracellular trap (MCET) formation. MCETs possibly contain antimycobacterial mediators, such as β-hexosaminidase and LL-37. **(C)** Finally, the MCs crosstalk with other immune cells [e.g., neutrophils, dendritic cells (DCs), and macrophages (Mφ)] contribute to antimycobacterial immunity. Although further experimental evidence is needed to prove the hypothesis, MCs seem to play a role in orchestrating tuberculosis granuloma formation and maintenance.

Since little is known about the MC contribution to TB pathogenesis, this review summarizes the MC strategies used in bacterial defense as well as potential and reported interactions occurring between *Mtb* and MCs (Figure 1).

## MC Ontogeny and Functions

Mast cells originate from pluripotent CD34^+^ and CD117^+^ hematopoietic stem cells and migrate as progenitors to various tissues where they mature influenced by the local microenvironment (15, 16). MCs are mainly located in the skin (~12,000/mm^3^) and mucosa including the lungs (~500–4,000/mm^3^) (10).

Mast cells express various PRRs (e.g., TLRs and CD48), complement receptors, and Fc receptors which upon engagement induce cell activation, degranulation, or both (17). Thereby, MCs release a great variety of pro- and anti-inflammatory pre-stored and *de novo* synthesized mediators such as histamine, heparin, tryptase, chymase, PGD_2_, LTC_4_/D_4_, chemokines as CCL1, CCL2, CCL4, CCL7, CCL12, CCL17, CXCL5, and CXCL8, and cytokines including IL-4, IL-3, IL-5, GM-CSF, IL-6, IL-13, IL-12, IFN-γ, TGFβ1, and TNF-α (18). This wide variety of products and their rapid release (in min/s) make MCs important modulators of inflammatory responses.

## MC Involvement in Early Mycobacterial Infection

Although interactions between MCs and *Mtb* have been reported (Figure 1), the role of MCs in TB pathogenesis remains unclear. MC involvement in mycobacterial immunity was first observed in guinea pigs using electron microscopy. Shortly, after intratracheal infection with *Mtb*, a significant increase in MCs was detected in guinea pig lungs (19). A later study demonstrated that the number of MCs in mice lungs increases by ~23% after 15 days of *Mtb* exposure (20).

### Bacillary Recognition

Rat peritoneal MCs (rPMCs) recognize *Mtb via* CD48 that is a glycosyl phosphoinositol-anchored cell surface protein (Figure 1A). Incubation with increasing concentrations of anti-CD48 antibodies together with *Mtb* correlate with a proportional decrease in histamine release (21). Previous work has shown that CD48 recognizes FimH protein expressed by fimbriated bacteria (such as *Escherichia coli* and *Staphylococcus aureus*) resulting in MC degranulation (12, 22, 23), raising the question of how precisely CD48 recognizes *Mtb*, which is not known to be fimbriated (24).

TLRs are a key receptor family implicated in pathogen recognition (22). Carlos and colleagues found MC TLR2 to be relevant in *Mtb* recognition (Figure 1A) (9). The transfer of TLR2^+/+^ MCs into TLR^−/−^ *Mtb*-infected mice showed an increase of cytokine release and cell recruitment, suggesting MC TLR2 as key receptor upon mycobacterial challenge (9). MCs also express TLR4 that serves as a mannose receptor in complex with soluble CD14. In the absence of CD14, high concentrations of TLR4 ligands are required for MC–TLR4 activation (25–27). Presently, it is unclear if TLR4 (or other TLRs) are involved in *Mtb* recognition and if the TLR4–CD14 complex is necessary to trigger MC functions. Besides the TLRs and CD48, the CR3, CR4, C3aR, and C5aR complement receptors and the FcεRI, FcγRI, FcγRII, and FcγRIII receptors mediate MC responses to other bacteria, but it is as yet unknown if they are involved in MC–*Mtb* interactions (12, 22, 28).

### Bacillary Binding and Uptake

Muñoz and colleagues suggested that *Mtb* is internalized by MCs *via* lipid rafts (Figure 1A). Cholesterol depletion of the rat basophilic leukemia cells (RBL-2H3) reduced internalization of mycobacteria. Interestingly, once internalized, mycobacteria survived intracellularly for 4 days after which most of the infected MCs (70%) had undergone lysis. These findings implicate MCs as a reservoir for *Mtb* (29). However, still unclear are the requirements and dynamics of *Mtb* internalization by MCs since few studies have demonstrated it experimentally and all were performed in animal models (21, 29). It is therefore important that the *Mtb* internalization is demonstrated in human MCs, together with the underlying mechanisms of internalization (aside from lipid rafts) and the intracellular compartments in which *Mtb* may reside. Published data describing MC interactions with other bacteria may guide the design of these investigations. For example, *Salmonella typhi* is taken up by complement receptors while *E. coli* is attached and internalized by FimH to the MC surface (30). Furthermore, preincubation of human MCs with IFN-γ increases the membrane attachment of *S. aureus* (31). Recently, the MC phagosome was demonstrated to interact with NOD-like receptors thus modulating cytokine production (32), and indicating that MC phagocytosis acts as an inducer of other MC activities.

### Bacterial-Induced Mediator Release

Few studies have characterized the *Mtb-*induced MC production of soluble mediators of inflammation. Muñoz et al. reported that after stimulation with *Mtb*, rPMCs released *de novo* synthesized TNF-α and IL-6, followed by secretion of histamine and β-hexosaminidase. In addition, specific *Mtb* antigens [MPT-63 and early secretory antigenic target 6 (ESAT-6)] have also been shown to induce rPMCs to release TNF-α, IL-6, histamine, and β-hexosaminidase (Figure 1) (21).

#### Cytokines and Chemokines

Cytokines and chemokines released by MCs contribute to protective immunity in the context of bacterial infections. For example, it has been shown that MC depletion reduces TNF-α (important in mycobacterial granuloma maintenance) concentrations in bronchoalveolar lavage (BAL) of *Bordetella pertussis*-infected mice (33). Furthermore, following infection with *Streptococcus pneumoniae*, increased TNF-α concentrations in the BAL and MC numbers in the lung correlated with protection (34). Similarly, MC-derived IL-6 produced during *Klebsiella pneumoniae* challenge was observed to improve mouse survival by promoting neutrophil recruitment and intra-neutrophil killing (35), the importance of which in mycobacterial immunity is being increasingly recognized (36). MCs also produce a wide variety of soluble mediators potentially relevant to mycobacterial immunity, including IL-13, IL-12, IL-6, IL-4, TNF-α, CCL5, CXCL2, CCL7, and CCL2, following infection with *Streptococcus equi* (37). Since the cocktail of soluble mediators produced by MCs appears dependent on the specific pathogen, a comprehensive proteomic assessment of mediators produced by MCs in response to *Mtb* is needed.

#### Antimicrobial Peptides (AMPs)

Antimicrobial peptides kill pathogens by forming pores in cytoplasmic membranes; defensins and cathelicidins are the most studied (38). Early in infection, cathelicidins promote phagocytosis, upregulate the expression of costimulatory molecules in DCs and stimulate Th1 cytokine production, while later in the course of the disease they inhibit the production of pro-inflammatory molecules (39). Although little is known regarding the AMP repertoire that MCs may release, MC supernatants reduce bacterial burden (40, 41). Cathelicidin LL-37 is expressed in human dermal skin MCs while the respective murine homolog cathelicidin-related AMP (CRAMP) is produced by bone marrow-derived MCs (BMMCs). Upregulation of CRAMP expression by LPS reduces group A streptococcus extracellular titers (42), while MC-derived LL-37 promotes clearance of *Enterococcus faecalis* (43). Finally, β-hexosaminidase, which is released by MCs after degranulation, was observed to exhibit antimicrobial activities upon intracellular *Listeria monocytogenes* infection (39) and mMCP-6, a mouse tryptase, was essential for *K. pneumoniae* clearance in mMCP-6^−/−^ mice (44). Thus, antimycobacterial molecules are likely to be secreted by MCs upon *Mtb* exposure (Figure 1B).

#### MC Degranulation and Histamine Release

Mast cell degranulation in *Mtb*-infected mice is associated with a decrease in leukocytes, neutrophils, mononuclear cells, IL-1β, TNF-α, MIP-2, IL-12, IFN-γ, and MCP-1 (20). Histamine is released during MC degranulation. Carlos and colleagues used histamine-deficient C57BL/6 mice to investigate the role of histamine in *Mtb* infection, which is detectable in high concentrations 28 days after *Mtb* infection. Mice lacking histamine showed decreased neutrophils numbers, as well as TNF-α and IL-6 levels in lung tissue, while IL-12 and IFN-γ concentrations were increased. Furthermore, the histamine-deficient lungs showed lymphocytic infiltration with an increase in the number of CD4^+^ T cells that correlated with reduced bacterial growth (45). Taken together, these findings suggest that MC degranulation may have a complex role in modulating the inflammatory response to *Mtb*. It would be important to investigate the redundancy of these pathways in *Mtb* infection using *in vivo models*, as well as determine whether MCs are the source of histamine in this context, which may indicate novel therapeutic avenues.

## MC Extracellular Traps

The formation of ETs, named ETosis, is a type of cell death characterized by release of DNA (46). ETosis differs from apoptosis and necrosis since it lacks DNA fragmentation, disruption of the nuclear envelope, absence of phosphatidylserine in the outer membrane and caspase-independent activation (47). The formation of MCETs upon cell stimulation relies on ROS production by MCs that in turn promotes nuclear envelope disruption and release of DNA together with granular components with antimicrobial properties. The DNA backbone in combination with histones, proteases, and AMPs (39, 48) forms physical traps that catch and expose pathogens to high concentrations of antimicrobial molecules (Figure 1B) (48, 49).

A human mast cell line (HMC-1), showed ET formation upon *L. monocytogenes* infection. This Gram-positive bacterium was shown to promote disruption of the nuclear envelope followed by an increase in ROS production. Interestingly, the presence of β-hexosaminidase in the traps was observed to have an antimicrobial activity to intracellular *L. monocytogenes* (39). *E. faecalis* was also found to induce MCET after 3 h of incubation with BMMCs. However, the level of MCET observed was lower compared with the one promoted by other bacteria (43). This was possibly due to the low multiplicity of infection (MOI) used in this study (MOI 1:1). *Streptococcus pyogenes* induced MCET after infection (MOI 25:1) in HMC-1 cells exhibiting cathelicidin LL-37, histones, and tryptase in the traps and in murine BMMCs displaying tryptase and histones (50). This suggests that high bacterial burden promotes MCET activation. Interestingly, besides bacterial stimulation, IL-12 and IL-1β were found to induce MCET containing IL-17 after the stimulation of MCs from skin explants of patients with psoriasis (51).

### *Mycobacterium*–MCET

*Mycobacterium tuberculosis* induces neutrophil ETs (NETs). However, Ramos-Kichik et al. have reported that although mycobacteria induce the formation of NETs, which include elastase and histones, the AMPs contained in the NETs are unable to kill mycobacteria (52). Three hour incubation of human neutrophils with the virulent *Mtb* and the less virulent *Mycobacterium canettii* showed that both mycobacteria were entrapped in NETs; however, neither *Mtb* nor *M. canettii* were killed. In fact, mycobacteria were not eliminated even at low bacterial concentrations (MOI 0.1:1), nor did NETs restrict ongoing mycobacterial replication.

Virulent *Mtb* secretes ESAT-6 and CFP-10 (10-kDa culture filtrate protein) through the ESX-1 secretory system. Both factors are important for the pathogenic intracellular pore-forming activities and phagosomal subversion observed in the early phase of TB (53). Interestingly, ESAT-6 has been shown to induce extracellular NETs by Ca^+^ influx (54). In addition, *Mtb* can induce ETs in human macrophages (Mφ) *via* the ESX-1 system, which is enhanced by IFN-γ (55). By contrast, in highly infected Mφ, it has been reported that after IFN-γ initiates necrosis without NETosis (56). This information suggests that virulent mycobacteria may actively promote NET formation to achieve their own ends of persistence, raising the hypothesis that MCETs may also be involved (Figure 1B). Although mycobacteria–MCETs have not been demonstrated, MCs produce a large repertoire of immunomodulatory mediators that are known to be contained in traps. Therefore, it is important that future studies investigate the mycobacteria induction of MCETs and the inclusion of antimycobacterial mediators.

## MC Immune Crosstalk and the Mycobacterial Granuloma

Studies involving a broad array of bacterial pathogens have demonstrated the important role of MCs in promoting recruitment, maturation, and bactericidal activity of Mφ, DCs, and neutrophils (Table 1). However, the potential roles of MCs in modulating the delicate orchestration of immune crosstalk in mycobacterial immunity have not yet been described (Figure 1C). MCs could easily coordinate granuloma formation and maintenance. In support of this notion, Taweevisit and Poumsuk reported a correlation between MC numbers and granuloma formation (57). Briefly, 45 lymph nodes from patients with TB lymphadenitis were analyzed to determine the frequency of MCs in the granulomatous region. The authors observed that the number of MCs positively correlated with the number of granulomas. A similar correlation was found between multinucleated giant cells and MCs in the lymph nodes (57). Similar studies have been performed using skin biopsies of patients suffering with leprosy (*Mycobacterium leprae*) (58). Lepromatous leprosy (disseminated disease with high bacillary load) was associated with the lowest dermal MC density compared with paucibacillary and localized tuberculous leprosy. This suggests that MC functions may have a role in driving a differential susceptibility to these polar forms of leprosy, an as yet poorly explained clinical phenomenon (59). Furthermore, the higher MC numbers located around granulomas in the tuberculous group were considered to be indirect evidence of the role of MCs in activating the immune response to *M. leprae* infection. Interestingly, numerous MCs were found in the highly fibrotic dermal area and in the epineurial layer of lepromatous leprosy lesions, suggesting that MCs could be involved in the induction of fibrosis, including fibrotic leprosy neuritis (60).

**Table 1:** MCs: immune cell crosstalk in antibacterial immunity.

Cell target	MC function	Mediator	MC type (mouse)	Bacteria/model	Technique	Reference	Open questions
Mφ	Inhibition of internalization and intracellular growth	IL-4	BMMCs	*Francisella tularensis* LVS	*In vitro* coculture	(41)	Do MCs promote intracellular *Mtb* killing in Mφ?
	Trogocytosis, caspase I expression in Mφ and FcεRI, and OX40L upregulation in MCs	FcεRI-encompassed vesicles	BMMCs	*F. tularensis* LVS	*In vitro* coculture	(61)	

Neutrophil	Recruitment and activation	TNF-α	Peritoneal	*Listeria monocytogenes*	MC-depleted BALB/c mice	(62)	Do MCs contribute to neutrophil recruitment? Do MCs promote intracellular Mtb killing in neutrophils? Is MC-derived TNF-α relevant in granuloma maintenance?
	Recruitment	?	Intestinal	*Clostridium difficile* toxin A	MC^−/−^ mice/reconstitution	(63)	
	Killing	IL-6	BMMCs	*Klebsiella pneumoniae*	IL-6-deficient mice	(35)	
	Recruitment	?	Skin	*Pseudomonas aeruginosa*	MC^−/−^ mice/reconstitution	(64)	
	Recruitment	MC TLR2	BMMCs	*Mycobacterium tuberculosis*	TLR2^−/−^ mice reconstitution with TLR^+/+^ MCs	(9)	

DC	Recruitment to the site of infection and migration to DLNs	TNF-α E-selectin	BMMCs	*Escherichia coli* (urinary tract infection)	MC^−/−^ mice/reconstitution	(65)	Do MCs contribute to DC recruitment? Do MCs modulate DCs-induced Th1 polarization? Do MCs enhance DC functions upon Mtb challenge?
	Maturation and Th polarization	IL-12 IFN-γ IL-6 TGF-β	Peritoneal	LPS	*In vitro* coculture	(66)	

*MC modulation upon bacterial stimulation*.

*LVS, live vaccine strain; MCs, mast cells; Mφ, macrophages; DCs, dendritic cells; BMMCs, bone marrow-derived MCs; DLNs, draining lymph nodes; LPS, lipopolysaccharide; FcεRI, high-affinity receptor for IgE*.

### MC-Derived Soluble Mediators and Mycobacterial Granuloma Maintenance

Mast cell-derived LL-37 and CRAMP are bactericidal for *Mtb* (67). Ramos-Espinosa and colleagues reported that the administration of adenovirus encoding the human cathelicidin LL-37 (AdLL37) and TNF-α (AdTNFα) had a protective role in inducing granuloma maintenance, and thus TB disease reactivation (68). These observations suggest that MCs and MC mediators in particular are involved in granuloma maintenance (Figure 1C). Similarly, von Stebut and colleagues reported that upon encounter with pathogens MCs release pre-stored TNF-α that induces neutrophil recruitment to the site of granulomatous inflammation. This was followed by the release of neutrophil-derived MIP-1α/β and MIP-2 chemokines both responsible for Mφ recruitment. Lack of this immediate pre-stored TNF-α release delayed Mφ recruitment and granuloma formation (69).

The study by Carlos et al. discussed earlier also described that during *Mtb* infection, TLR2 engagement induces cytokine release in the lung; as observed in a reconstitution murine model after 60 days of infection (9). The transfer of TLR2^+/+^ MCs into TLR^−/−^ *Mtb*-infected mice showed diminished lung bacterial growth and an increase of TNF, IL-6, IL-1β concentrations, and neutrophil and mononuclear cell recruitment resulting in the restoration of granuloma formation (9). Therefore, MCs may be involved not only in the early but also in the late phase of infection. Furthermore, MC-derived IL-6 and TNF-α in this phase of infection may contribute to granuloma maintenance (9). The precise contribution of MCs in mycobacterial granuloma maintenance remains an important open question, and *in vivo Mtb* infection models combined with MC reconstitution experiments may yield critical insights into this area.

## Conclusion

Tuberculosis is a highly contagious infectious disease caused by *Mtb* which infects billions, and kills millions of people worldwide. Although many efforts have been made to reduce TB mortality, the infection remains one the most important threats to human health. MCs are key lung resident immune sentinels that contribute to antibacterial immunity and are likely to play a key role in TB pathogenesis. A potential important and unique function of MCs is the crosstalk with other immune cells to orchestrate multiple effector functions, which may contribute to granuloma formation and maintenance. We have highlighted the potential roles MCs may play during TB that once addressed could inform the design of novel therapeutic strategies.

## Author Contributions

KG-R and SB-P conceived, wrote, designed, and coordinated the manuscript. AG, MA-R, and RH-P provided helpful discussions and edited the manuscript. All the authors read and approved the final manuscript.

## Conflict of Interest Statement

The authors declare that the research was conducted in the absence of any commercial or financial relationships that could be construed as a potential conflict of interest.
